# Correlations between prescription of anti-hypertensive medication and mortality due to stroke

**DOI:** 10.1186/1471-2261-12-15

**Published:** 2012-03-12

**Authors:** Renata Papp, Albert Csaszar, Edit Paulik, Sandor Balogh

**Affiliations:** 1 National Institute of Primary Health Care, (84-88 Jasz Str.), Budapest 1135, Hungary; 2 Department of Public Health, Faculty of Medicine, University of Szeged (10 Dom ter), Szeged 6720, Hungary; 3 2nd Department of Medicine, Military Hospital State Health Centre, (109-111 Podmaniczky Str.), Budapest 1062, Hungary

## Abstract

**Background:**

One of the most important risk factors for stroke is hypertension. A number of studies have attempted to identify the most effective anti-hypertensive therapeutic group for stroke prevention. Using an epidemiologic approach we aimed to find correlations based on Hungarian data on stroke-mortality and on prescription routine of anti-hypertensive therapeutics in three different counties, showing significant difference in stroke mortality.

**Methods:**

We have used the official yearly reports on stroke-mortality for the period 2003-2008. Based on the significant differences in the change in mortality due to stroke three counties were selected: Baranya, Bekes and Hajdu-Bihar. The usage of antihypertensive therapeutic groups was analyzed. The correlation of stroke mortality difference and different antihypertensive treatment habits was analyzed by using normality test, time series analyses, correlation coefficient, paired samples test, one sample test and chi-square test.

**Results:**

For the year 2003 stroke-mortality standardized with the county population number was highest in county Bekes, followed by county Baranya and county Hajdu-Bihar. For each year stroke mortality has shown significant (p < 0.0001) difference between the three counties and the ranking/order of the counties has been preserved over time. During the period of our study, an increase in the number of days of treatment was observed for most of the anti-hypertensive drugs listed. We have observed that the increased use of high-ceiling diuretics resulted in a mortality advantage, and the reduction in use of calcium channel blockers with direct cardiac effect had negative consequences.

**Conclusions:**

The authors acknowledge that by limiting the study to three counties the findings cannot be generalized to the whole Hungarian population. Two trends can still be identified:

i) increased number of days of treatment (and therefore the probable use) of high-ceiling diuretics is associated with reduction in mortality due to stroke and its immediate complications; ii) reduction in the use of non-dihidropiridin CCBs does not seem justified, as their use appears to be advantageous in stroke prevention. Authors put emphasis on the importance of the adherence of the patients to the preventive therapies. Health care professionals could provide an important added value to the life long preventive therapies by improving the compliance of their patients, giving personalized care and advice.

## Background

The widespread reduction in cardio-vascular mortality in the recent past can be ascribed to a number of favorable changes. Out of a multitude of contributing factors we have previously documented the favorable role of the steady increase in the prescription of cardio-metabolic therapies [1]. That study described a significant correlation between the increase in prescription of three therapeutic groups (anti-diabetic, anti-lipidemic and anti-hypertensive) and the reduction in mortality due to stroke and acute myocardial infarction (AMI). Out of the above mentioned cardio-metabolic therapeutic groups the most noteworthy increase in prescriptions was observed for anti-hypertensive drugs. The goal of our present study is to focus specifically on the correlation between anti-hypertensive prescriptions and mortality due to stroke. During our study we review data collected from three Hungarian counties and compare these data over a period of 6 years.

One of the most important risk factors for stroke is hypertension. It has been shown that blood pressure levels higher than 115/75 mmHg display a linear correlation between blood pressure level and mortality and also morbidity due to stroke [2,3]. In the 40 to 70 years age group it was shown that a 20 mmHg increase in systolic blood pressure or a 10 mmHg increase in diastolic blood pressure double the risk of stroke [4]. Therefore, reducing blood pressure levels could be one of the most effective mechanisms for decreasing the incidence of stroke and mortality due to stroke.

All anti-hypertensive therapeutics reduce the risk of emergence of stroke and coronary disease and their efficacy correlates with the degree of decrease of systolic blood pressure levels [5,6]. A number of studies have attempted to identify the most effective anti-hypertensive therapeutic group for stroke prevention. Meta-analysis studies have shown that the risk of stroke is reduced with calcium-channel blockers (CCBs) more, than the expected levels for a given reduction in the systolic blood pressure level while beta-blockers reduce stroke-risk less, than the other anti-hypertensive therapeutic drugs [6,7].

Using an epidemiologic approach we aimed to find correlations based on Hungarian data on stroke-mortality and on prescription routine of anti-hypertensive therapeutics.

## Methods

We have used the official yearly reports on stroke-mortality for the period 2003-2008 published by the Hungarian Central Statistics Office (KSH) [8]. The mortality statistics provided by KSH are based on death certificate records and sum up the main diagnoses for cause of death. Diagnoses used are defined according to the International Classification of Diseases Tenth Revisions (ICD-10). The yearly reports contain the mortality data of Hungarian counties normalized for the population of each county. Accordingly, our use of the term stroke-mortality includes fatal stroke and death due to direct complications of stroke.

Following a preliminary analysis of stroke mortality changes per 100 000 inhabitants in different counties, three counties were selected: Baranya, Bekes and Hajdu-Bihar based on the significant differences in the change in mortality due to stroke.

Of the 19 counties in Hungary Hajdu-Bihar had the lowest stroke mortality in the two years: 132.3/100 000 inhabitants in 2003 and 83.2/100 000 inhabitants in 2008. Bekes showed the 2nd highest mortality (200.5/100 000 inhabitants) in 2008 but compared to the first highest (Zala county with 212.4/100 000 inhabitants) showed less improvement (36.5 versus 57.4) during the 6 years of examination. Baranya had the 3rd lowest stroke mortality rate (124.8/100 000 inhabitants) in 2008 (without considering Hajdu-Bihar which was already included), but started from a higher level (173,5/100 000 inhabitants) in 2003, so the improvement was higher (48.7) in this county than in Pest county (32.1) and the capital Budapest (43.7). So we ended up with three counties: one (Hajdu-Bihar) with the lowest stroke mortality rates in 2003 and 2008, another (Bekes) with the lowest improvement among the counties with high mortality rate in 2008, and one (Baranya) with the best improvement among the ones with lowest mortality rate in 2008. In order to find out differences in the anti-hypertensive treatment, and possible association with the stroke mortality changes, we analyzed these three counties with different specific patterns of change in stroke mortality.

The anti-hypertensive therapies we have studied belong to the following ATC (anatomical, therapeutic and chemical) groups: centrally acting antiadrenergic agents (ATC: C02A); peripherally acting antiadrenergic agents (ATC: C02C); low-ceiling diuretics, thiazides (ATC: C03A, C03B); high-ceiling diuretics (ATC: C03C); potassium-sparing agents (ATC: C03D); beta blocking agents (ATC: C07A); selective calcium channel blockers with mainly vascular effects (ATC: C08C); selective calcium channel blockers with direct cardiac effects (ATC: C08D); plain ACE-inhibitors (ATC: C09A), plain angiotensin II antagonists (ATC: C09C) (see Additional file 1).

The usage of these therapeutic groups in each county was obtained from data provided by the Hungarian National Health Insurance Fund (OEP) yearly reports for 2003-2008 [9] compiled yearly and per county from the dispensing records of all retail pharmacies in Hungary. OEP reports express the use of therapeutic agents as number of days of treatment (DOT) per ATC group for each county. DOT values take into account differences in drug strength and formulation based on the common denominator of the daily defined dose (DDD) defined by the WHO. Due to the character of source data we studied the total usage of therapeutics at county level, corrected to the county population. Reports do not contain data on combination therapies and do not include information on physician characteristics, patient-level data (e.g. age, sex, co-morbidities, or concomitant medications dispensed), or indications for use. Data were not age and sex standardized.

Both databases [8,9] are freely accessible; no specific approval was required for use.

The relationship between standardized yearly rates of mortality per 100 000 inhabitants in each county and the yearly days of treatment per each therapeutic group corrected to the county population was examined. This analysis was designed to assess the association between the changes in prescriptions and the changes in mortality rates as a function of time (years). We also determined the correlation coefficients between the number of days of treatment and mortality rates. All of the computations were solved using MedCalc and Zaitun Times Series statistical software. The applied statistical methods were: Normality test, time series analyses, correlation coefficient, paired samples test, one sample test and chi-square test.

## Results

**Figure 1:**
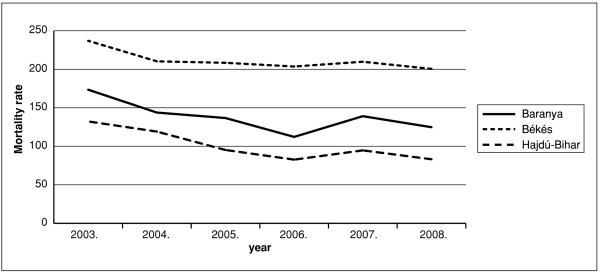
**Change of mortality rate (/100,000 inhabitants) in the three counties during the period 2003**-**2008**.

**Figure 2:**
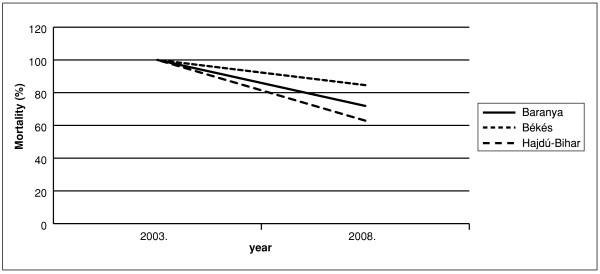
**Stroke mortality decrease expressed as a percentage of the 2003-value**.

**Figure 3:**
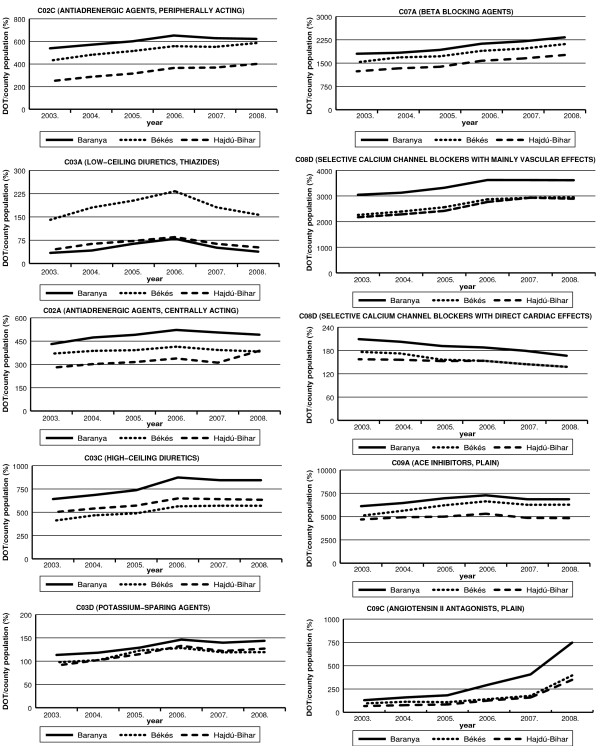
**Change in the days of treatment in the three counties (analysed ATC groups on separate diagrams)**.

For the year 2003 stroke-mortality standardized with the county population number was highest in county Bekes, followed by county Baranya and county Hajdu-Bihar (Figure 1). During the following years mortality decreased in each county, except for the uniform increase of small magnitude observed in 2007. For each year stroke mortality has shown significant (p ≤ 0.0001) differences between the three counties and the ranking/order of the counties has been preserved over time. In all three counties the rate of mortality decrease due to stroke was different, the most marked change being observed in county Hajdu-Bihar (Figure 2). During the period of our study, an increase in the number of days of treatment was observed for most of the anti-hypertensive drugs listed (Figure 3).

Our analyses show a connection between the changes in the prescription of different anti-hypertensive drugs and the decrease over time of mortality due to stroke for only two of the eleven studied therapeutic groups (see Additional file 1). The comparison of the number of days of treatment with high-ceiling diuretics (ATC: C03C) between county Baranya and county Bekes showed a bigger increase in diuretics prescription in county Baranya and a more accentuated decrease in stroke-mortality was recorded in the same county (see Additional file 2). In the comparison of counties Bekes and Hajdu-Bihar in regard to the decrease in the use of selective calcium channel blockers with direct cardiac effects we found that in county Hajdu-Bihar, where the drop in these prescriptions was smaller, stroke-mortality remained at lower levels (see Additional file 2). Additional file 3 shows the results of the analysis between counties for each group of antihypertensive treatment highlighting the significant difference presented in Additional file 2. In conclusion, while in the first case the increased use of high-ceiling diuretics was associated with a mortality advantage, in the latter case the reduction in use of C08D-group drugs was linked to negative consequences.

## Discussion

Reduction of high blood pressure is one of the most important preventive interventions concerning primary (first) stroke. Blood pressure reduction diminishes coronary risk as well and the scale of blood pressure decrease correlates with the efficacy of prevention [10].

Former studies have demonstrated that all the therapeutic drugs used for the treatment of systolic-and-diastolic hypertension and for the treatment of isolated systolic hypertension reduce stroke incidence independently of the specific medication [5,6]. According to the BPLTTC meta-analysis, which used data from 29 randomized, controlled studies, the risk of stroke can be reduced by 30-40% by the use of any anti-hypertensive treatment [5]. Independently of the actual vascular risk-status of a patient a reduction by 10 mmHg of the systolic blood pressure, or by 5 mmHg of the diastolic blood pressure can reduce the number of coronary events by approx. 25% and of stroke by approx. 30% [6].

It is widely recognized that differences exist in efficacy amongst different groups of anti-hypertensive drugs. A meta-analysis of nine studies showed a 10% -although not statistically significant- advantage of calcium-channel blockers (CCBs) over other therapeutic group [11]. The formerly mentioned BPLTTC analysis has documented that CCBs have a more favorable effect compared to ACE-inhibitors, diuretics or beta-blocking agents. A later meta-analysis [12] which reviewed 13 studies, comprising in aggregate more than 100,000 patients, found that CCBs reduced the incidence of stroke to a greater extent than the other anti-hypertensive drugs. Notably, the CCB-mediated decrease in stroke-incidence did not directly correlate with their anti-hypertensive effect. A possible explanation for this observation could be that CCBs can mitigate the excessive influx of calcium into neurons, thereby diminishing neuronal necrosis in ischemic brain regions. The meta-analysis was able to differentiate between the CCBs with mainly vascular effects and CCBs which have a direct cardiac effect. CCBs with mainly vascular effects were 10% more effective than other anti-hypertensive drugs, while CCBs with direct cardiac effects were 8% more effective. The later was not statistically significantly different from the efficacy of the other therapeutic groups.

According to the investigators, this result is due to the inclusion into the meta-analysis of the CONVINCE study, which was ended prematurely based on trend-like results which were emerging during the study, and which casts doubts upon the utilization of these results [12].

The INVEST [13] and VHAS [14] studies have reported no differences when comparing the efficacy of verapamil--a CCB with direct cardiac effects--with beta-blocking agents [13] or with diuretics [14]. In contrast, the NORDIL study [15] demonstrated a stronger effect of the CCB diltiazem compared to both diuretic- and beta-blocking-therapies with regards to stroke occurrence. This difference in efficacy could possibly be explained by the extent of blood pressure reduction, which was 3 mmHg greater using diltiazem. Unfortunately, there are no available studies directly comparing the effects on stroke incidence for drugs belonging to the two different groups of CCBs.

Non-dihidropiridin CCBs might show an advantage over CCBs with mainly vascular effects due to their additional actions such as their ability to reduce heart rate (with consequently diminished sympathetic nervous system activation) and their ability to revert left ventricular hypertrophy.

## Conclusions

According to the results, the use of CCBs with direct cardiac effects is associated with a favorable influence on stroke-mortality in the three Hungarian counties examined. Our study did not aim to analyze a relationship between drug usage and the incidence of stroke but drug usage and the mortality connected to stroke. The stroke-mortality source data for this study reports mortality due to the acute phase of stroke (fatal stroke) and mortality due to direct complications of stroke as an aggregate value. Stroke-related mortality in particular is influenced by the extent of cerebral damage however cardiac status is also a determining factor. Thus, non-dihidropiridin CCBs could positively influence both of these factors, by reducing the degree of cerebral damage and improving recovery as well as reducing cardiac effects by mitigating the activation of the sympathetic nervous system.

The positive correlation between the use of high-ceiling diuretics and decrease in stroke mortality can be explained mainly by the reduction in blood pressure levels, however a favorable effect on the associated heart insufficiency could also be a contributing factor.

Stroke-mortality is influenced by many factors and the data presented here are intended mainly to raise awareness to some of them. Life-style parameters/measurements and treatments of risk factors other than hypertension were not included. The overall cardiovascular risk factors of the Hungarian population are likely to have increased (obesity, diabetes mellitus) while leisure-time physical activity and smoking data did not change over the studied time frame. The lack of breakdown of these data on a county level made the authors consider association with anti-hypertensive treatments only, as the main influencing factor in stroke mortality. Improvement of the acute care of stroke patients could also have a positive influence on mortality rates. In the future a wider analysis of influencing factors could lead to more precise statements about causes of the differences experienced amongst counties.

It is reported in meta-analysis studies that CCBs reduce stroke occurrence to a greater extent than other anti-hypertensive agents, and this favorable effect likely extends to mortality due to stroke, both in its acute phase and post-stroke period. This favorable effect is probably independent of the CCBs dihidropiridin sub-classification.

The authors acknowledge that by limiting the study to three counties the findings cannot be generalized to the whole Hungarian population. Two trends can still be identified:

i) increased number of days of treatment (and therefore the probable use) of high-ceiling diuretics is associated with reduction in mortality due to stroke and its immediate complications; ii) reduction in the use of non-dihidropiridin CCBs does not seem justified, as their use appears to be advantageous in stroke prevention. Although, there are obvious limitations to the present study (use of aggregated mortality data; lack of data regarding combination therapies; lack of county-specific data on other risk factors such as lifestyle), our findings are in concordance with the results of the interventional studies, demonstrating the utility of this kind of assessment. Nevertheless choosing the appropriate medical treatment has to be accompanied by the personalized advice and regular control of the patients. The adherence to the life long therapies will give an added value to the preventive care and increase the quality of life of the chronically ill patients. The ways the different available resources are used by the health care system will result in different outcomes which can be identified among different geographical areas even in a relatively small country like Hungary. As the constraint of cost-effective usage of resources characterizes the actual health care policies we hope our findings could contribute to the health promotion and better quality of life of patients with chronic cardiovascular diseases.

## Competing interests

The authors declare that they have no competing interests.

## Authors' contributions

RP has made substantial contributions to conception, acquisition of the data on mortality and medication and their contribution to the changes in stroke mortality in the specific counties. SB has been involved in drafting the manuscript and revising it critically for important intellectual content. EP has been involved in the acquisition of data, analysis and interpretation of data. ACs has made substantial contributions to conception and design, has given final approval of the version to be published. All authors read and approved the final manuscript.

## Pre-publication history

The pre-publication history for this paper can be accessed here:

http://www.biomedcentral.com/1471-2261/12/15/prepub

## Supplementary Material

Additional file 1 **Table S1**. Active ingredients corresponding to the ATC groups analysed. Click here for file

Additional file 2 **Table S2**. Correlation between change in mortality and drug consumption. Click here for file

Additional file 3 **Table S3**. Analysis of correlation between change in mortality and drug consumption of all ATC groups included. Click here for file
