# Supplementary material for: Correlations between prescription of anti-hypertensive medication and mortality due to stroke
Source: BMC Cardiovasc Disord. 2012 Mar 12;12:15. doi: 10.1186/1471-2261-12-15 (PMC3323458; doi:10.1186/1471-2261-12-15)
Supplement: Additional file 1 — Table S1. Active ingredients corresponding to the ATC groups analysed. [file 1471-2261-12-15-S1.PDF]

Table 1. Active ingredients corresponding to the ATC groups analysed

ATC code	ATC group	Active ingredients registered in Hungary
C02A	antiadrenergic agents, centrally acting	moxonidine, methyldopa, guanfacine, rilmenidine
C02C	antiadrenergic agents, peripherally acting	urapidil, doxazosin, terazosin, prazosin
C03A	low-ceiling diuretics, thiazides	hydrochlorothiazide
C03B	low-ceiling diuretics, excluding thiazides	indapamide, clopamide, chlortalidone
C03C	high-ceiling diuretics	etacrynic acid, furosemide
C03D	potassium-sparing agents	spironolactone, eplerenone, triamterene
C07A	beta blocking agents	atenolol, nebivolol, metoprolol, metoprolol succinate, bisoprolol, esmolol hydrochloride, esmolol, carvedilol, celiprolol hydrochloride, propranolol, betaxolol, bopindolol, sotalol, pindolol
C08C	selective calcium channel blockers with mainly vascular effects	nifedipine, amlodipine, amlodipine besilate, nisoldipine, nitrendipine, felodipine, manidipine, lacidipine, lercanidipine, isradipine, nimodipine
C08D	selective calcium channel blockers with direct cardiac effects	diltiazem, verapamil, gallopamil
C09A	ace inhibitors, plain	quinapril, captopril, enalapril, perindopril, trandolapril, ramipril, cilazapril, lisinopril, fosinopril, perindopril tert-butylamine, benazepril, spirapril, imidapril, zofenopril calcium
C09C	angiotensin II antagonists, plain	valsartan, irbesartan, losartan, candesartan, irbesartan, telmisartan, olmesartan, olmesartan medoxomil, eprosartan
