# Supplementary material for: Correlations between prescription of anti-hypertensive medication and mortality due to stroke
Source: BMC Cardiovasc Disord. 2012 Mar 12;12:15. doi: 10.1186/1471-2261-12-15 (PMC3323458; doi:10.1186/1471-2261-12-15)
Supplement: Additional file 2 — Table S2. Correlation between change in mortality and drug consumption. [file 1471-2261-12-15-S2.PDF]

Table 2. Correlation between change in mortality and drug consumption

	C03C high-ceiling diuretics	
	BaranyaC03C_BékésC03C	
MortBaranya_MortBékés	–0,875	Correlation Coefficient
	0,0223	Significance Level p

	C08D selective calcium channel blockers with direct cardiac effects	
	BékésC08D_Hajdú-BiharBC08D	
MortBékés_MortHajdú-Bihar	–0,869	Correlation Coefficient
	0,0246	Significance Level p
