# Supplementary material for: Correlations between prescription of anti-hypertensive medication and mortality due to stroke
Source: BMC Cardiovasc Disord. 2012 Mar 12;12:15. doi: 10.1186/1471-2261-12-15 (PMC3323458; doi:10.1186/1471-2261-12-15)
Supplement: Additional file 3 — Table S3. Analysis of correlation between change in mortality and drug consumption of all ATC groups included. [file 1471-2261-12-15-S3.PDF]

Table 3. Analysis of correlation between change in mortality and drug consumption

		BéB01A_HaB B01A	BéC01D_HaB C01D	BéC02A_HaB C02A	BéC02C_HaB C02C	BéC03A_HaB C03A	BéC03B_HaB C03B	BéC03C_HaB C03C	BéC03D_HaB C03D	BéC07A_HaB C07A	BéC08C_HaB C08C	BéC08D_HaB C08D	BéC09A_HaB C09A	BéC09C_HaB C09C	BéC10A_HaB C10A
MortBé_MortHaB	Correlation Coefficient	0,56	0,198	-0,235	-0,151	0,404	0,737	0,089	-0,475	-0,162	-0,119	-0,869	0,784	-0,345	0,745
	Significance Level P	0,2479	0,7065	0,6538	0,7754	0,4273	0,0946	0,8662	0,3406	0,7598	0,8218	0,0246	0,0652	0,5031	0,0891
	n	6	6	6	6	6	6	6	6	6	6	6	6	6	6

		BaB01A_HaB B01A	BaC01D_HaB C01D	BaC02A_HaB C02A	BaC02C_HaB C02C	BaC03A_HaB C03A	BaC03B_HaB C03B	BaC03C_HaB C03C	BaC03D_HaB C03D	BaC07A_HaB C07A	BaC08C_HaB C08C	BaC08D_HaB C08D	BaC09A_HaB C09A	BaC09C_HaB C09C	BaC10A_HaB C10A
MortBa_MortHaB	Correlation Coefficient	0,488	-0,271	0,216	-0,404	0,362	0,447	0,207	0,714	0,758	-0,194	-0,247	0,347	0,371	0,295
	Significance Level P	0,3266	0,6041	0,6809	0,4271	0,4808	0,3736	0,6944	0,5614	0,0808	0,7132	0,6367	0,4998	0,4689	0,5698
	n	6	6	6	6	6	6	6	6	6	6	6	6	6	6

		BaB01A_BéB 01A	BaC01D_BéC 01D	BaC02A_BéC 02A	BaC02C_BéC 02C	BaC03A_BéC 03A	BaC03B_BéC 03B	BaC03C_BéC 03C	BaC03_-BéC03D	BaC07A_BéC 07A	BaC08C_BéC 08C	BaC08D_BéC 08D	BaC09A_BéC 09A	BaC09C_BéC 09C	BaC10A_BéC 10A
MortBa_MortBé	Correlation Coefficient	-0,594	0,512	-0,632	0,157	0,681	-0,446	-0,875	-0,24	-0,043	0,116	-0,437	0,607	-0,381	0,296
	Significance Level P	0,2137	0,2995	0,178	0,7661	0,1365	0,3751	0,0223	0,6476	0,936	0,8262	0,3865	0,2017	0,4555	0,5684
	n	6	6	6	6	6	6	6	6	6	6	6	6	6	6

Pearson correlation coefficient

Ba = Baranya county
Bé = Békés county
HaB = Hajdú-Bihar county
